# The relationships between women’s reproductive factors: a Mendelian randomisation analysis

**DOI:** 10.1186/s12916-022-02293-5

**Published:** 2022-03-24

**Authors:** Claire Prince, Gemma C. Sharp, Laura D. Howe, Abigail Fraser, Rebecca C. Richmond

**Affiliations:** 1 MRC Integrative Epidemiology Unit, University of Bristol, Bristol, UK; 2 Population Health Sciences, Bristol Medical School, University of Bristol, Bristol, UK

**Keywords:** Mendelian randomisation, Reproductive factors, Genetic correlation, UK biobank

## Abstract

**Background:**

Women’s reproductive factors include their age at menarche and menopause, the age at which they start and stop having children and the number of children they have. Studies that have linked these factors with disease risk have largely investigated individual reproductive factors and have not considered the genetic correlation and total interplay that may occur between them. This study aimed to investigate the nature of the relationships between eight female reproductive factors.

**Methods:**

We used data from the UK Biobank and genetic consortia with data available for the following reproductive factors: age at menarche, age at menopause, age at first birth, age at last birth, number of births, being parous, age first had sexual intercourse and lifetime number of sexual partners. Linkage disequilibrium score regression (LDSC) was performed to investigate the genetic correlation between reproductive factors. We then applied Mendelian randomisation (MR) methods to estimate the causal relationships between these factors. Sensitivity analyses were used to investigate directionality of the effects, test for evidence of pleiotropy and account for sample overlap.

**Results:**

LDSC indicated that most reproductive factors are genetically correlated (*r*_g_ range: |0.06–0.94|), though there was little evidence for genetic correlations between lifetime number of sexual partners and age at last birth, number of births and ever being parous (*r*_g_ < 0.01). MR revealed potential causal relationships between many reproductive factors, including later age at menarche (1 SD increase) leading to a later age at first sexual intercourse (beta (*B*) = 0.09 SD, 95% confidence intervals (*CI*) = 0.06,0.11), age at first birth (*B* = 0.07 SD, *CI* = 0.04,0.10), age at last birth (*B* = 0.06 SD, *CI* = 0.04,0.09) and age at menopause (*B* = 0.06 SD, *CI* = 0.03,0.10). Later age at first birth was found to lead to a later age at menopause (*B* = 0.21 SD, *CI* = 0.13,0.29), age at last birth (*B* = 0.72 SD, *CI* = 0.67, 0.77) and a lower number of births (*B* = −0.38 SD, *CI* = −0.44, −0.32).

**Conclusion:**

This study presents evidence that women’s reproductive factors are genetically correlated and causally related. Future studies examining the health sequelae of reproductive factors should consider a woman’s entire reproductive history, including the causal interplay between reproductive factors.

**Supplementary Information:**

The online version contains supplementary material available at 10.1186/s12916-022-02293-5.

## Background

A woman’s reproductive life course includes her age at menarche and menopause, the age at which she starts and stops having children and the number of children she has, as well as the age she first has sexual intercourse and the number of sexual partners she has in her lifetime. Some of these reproductive factors have been identified as risk factors for chronic diseases, including breast cancer, respiratory disease and cardiometabolic diseases [1]. A younger age at menarche and older age at menopause were associated with an increased risk of breast cancer in one large meta-analysis [2], while having fewer children and a higher age at first birth (AFB) were positively associated with breast cancer risk in another [3]. Other studies have implicated age at menarche, AFB, number of still births and miscarriages, age at menopause and parity in relation to respiratory and cardiovascular disease [4–6]. One study found that later age at menarche was associated with a reduced risk of coronary artery disease [7]. Having any children and later AFB have been associated with a lower risk of lung cancer [8]. Older age at menarche and a shorter reproductive period have also been linked with a higher risk of chronic kidney disease [9, 10].

However, on the whole, studies have not considered a woman’s entire reproductive history and the potential interplay between reproductive factors. Understanding the inter-relationships between reproductive factors is important to correctly identify potential confounders (common causes of the exposure and outcome of interest) and mediators (factors that lie on the causal pathway between exposure and outcome). Information on multiple reproductive factors will provide useful additions to algorithms for predicting disease risk in women [1].

Evidence of association between age at menarche and menopause is inconsistent, with some studies reporting earlier age at menarche associated with earlier menopause [11–16], others showing the inverse association [17, 18] and some showing no evidence of this association [19–24]. While there is some evidence of an association between an earlier age at menarche and earlier AFB [25, 26], there is little evidence of the association between age at menarche and parity [26]. Another study has also investigated reproductive factors in relation to sexual history, suggesting a younger age at menarche is not a risk factor for younger age at first having sexual intercourse (AFS) [27]. Associations between reproductive factors could be reflective of causal relationships, or common genetic or non-genetic environmental causes, i.e. confounding.

Observational studies are prone to confounding bias as it is difficult to capture all confounders accurately. Mendelian randomisation (MR) is a method that assesses the causal relationship between an exposure and outcome by using genetic variants robustly associated with the exposure. MR is advantageous as it is less likely to be affected by confounding and reverse causation than standard multivariable regression analysis [28–30]. There have been an increasing number of genome-wide association studies (GWAS) of reproductive factors [31–33], which can be used to investigate genetic correlation (i.e. shared genetic causes) between these factors as well as whether relationships between reproductive factors may be causal using MR.

The present study aims to identify and clarify the nature of any relationships between women’s reproductive factors, by investigating their genetic overlap and the causal relationships between eight reproductive factors, including potential bidirectional effects where the temporal order between the traits is not clear.

## Methods

### UK Biobank

The UK Biobank study is a large population-based cohort of 502,682 individuals who were recruited at ages 37–73 years across the UK between 2006 and 2010. The study includes extensive health and lifestyle questionnaire data, physical measures and biological samples from which genetic data has been generated. The study protocol is available online, and more details have been published elsewhere [34]. At recruitment, the participants gave informed consent to participate and be followed up.

### Reproductive factors

The reproductive factors investigated in this study were age at menarche, age at menopause, age at first live birth, age at last live birth, number of live births, age first had sexual intercourse, lifetime number of sexual partners (at the time of assessment) and parous status (ever/never given birth at the time of assessment). In UK Biobank, these reproductive factors were derived from questionnaire responses at the baseline assessment; further details can be found in Additional file 1.

#### Phenotypic correlation

We calculated the correlation between reproductive factors using the Pearson correlation coefficient.

### GWAS

To identify genetic variants robustly related to each of the reproductive factors, we first performed a GWAS for each reproductive factor among women of European ancestry in the entire UK Biobank sample. Each GWAS was performed using the Medical Research Council (MRC) Integrative Epidemiology Unit (IEU) UK Biobank GWAS pipeline [35, 36]. BOLT-LMM was used to conduct the analysis in the GWAS pipeline [37], which accounts for population stratification and relatedness using linear mixed modelling. Genotyping chip and age were included as covariates. Genome-wide significant single nucleotide polymorphisms (SNPs) were selected at *p* < 5 × 10^−8^ and were clumped to ensure independence at linkage disequilibrium (LD) *r*^2^ < 0.001 and a distance of 10,000 kb using the TwoSampleMR package [35].

### Genetic correlation

Genetic correlations between the reproductive factors were calculated using LD score regression (LDSC) and the UK Biobank GWAS summary statistics [38, 39]. The regressions were performed using pre-computed LD scores for each SNP calculated based on individuals of European ancestry from using 1000 Genomes European data and are appropriate for use with European GWAS data [38]. These LD scores were filtered to HapMap3 SNPs as these are well-imputed in most studies [40]. SNPs found on chromosome 6 in the region 26 to 34MB were excluded. GWAS summary statistics were converted for LDSC regression using the munge_sumstats.py command from the command line tool “ldsc”, and LDSC was performed using the ldsc.py command.

### Mendelian randomisation

We conducted MR analysis using the “TwoSampleMR” R package [35], where the inverse variance weighted (IVW) method was used in the primary analysis to assess the causal relationships between pairs of reproductive factors. This method combines Wald ratios, calculated by dividing the SNP-outcome association by the SNP-exposure association, in a multiplicative random effect meta-analysis where the weight of each ratio is the inverse of the variance of the SNP-outcome association [41].

We assessed earlier-occurring reproductive factors as the exposure in relation to later-occurring factors (the outcomes), e.g. age at menarche was investigated as a potential cause of AFB but not vice versa. In some cases where there was no clear temporal ordering, we carried out analyses in both possible directions, e.g. between ever parous status and lifetime number of sexual partners. These cases are shown in Additional file 2: Table S1. Additionally, we investigated the effect of age at menopause on earlier-occurring factors: age at menarche, AFS and AFB to assess the effect of ovarian reserve, using age at menopause as a proxy.

All relationships tested by MR are shown in Additional file 2: Table S2, and GWAS estimates were standardised (mean = 0 and standard deviation (SD) = 1) prior to performing MR.

The IVW method makes a number of assumptions: that the genetic instruments are strongly associated with the exposure; do not share common causes, either genetic or other confounders such as population stratification, with the outcome; and are not pleiotropic, i.e. do not have an effect on the outcome through a pathway other than via the exposure [41]. We therefore performed a series of sensitivity analyses to evaluate the robustness of our results to these assumptions (see the “Evaluating MR assumptions” section).

In our primary analysis, we applied two-sample MR methods on a single large dataset, UK Biobank, which is advantageous over other methods due to the large sample size. In this analysis, the GWAS used for the exposure and outcome were both performed on women in the UK Biobank study, and therefore, the exposure and outcome samples overlap entirely. Large overlap in the sample(s) used to generate genetic variant-exposure and genetic variant-outcome associations can introduce bias in estimates obtained using two-sample MR [42]. In particular, sample overlap between the exposure and outcome samples may bias estimates towards the observational (and potentially confounded) exposure-outcome association and may lead to an overestimation of effects [42]. While it has been proposed that this approach of applying two-sample MR methods in a single sample may be performed within large studies with minimal bias introduced to the causal estimates by sample overlap [43], we performed a series of sensitivity analyses to evaluate the robustness of our results to this (see the “Assessing the impact of sample overlap” section).

### Evaluating MR assumptions

We evaluated the likelihood that MR assumptions were violated where we found evidence of effects in our primary analysis.

#### Instrument strength

The strength of the genetic instrument for each reproductive factor in the main IVW analysis was assessed using the mean *F* statistic, calculated based on the variance explained (*r*^2^) by the genetic instrument and sample size of the exposure [30].

#### Negative controls

We repeated our primary analysis for five “negative control” pairs of reproductive factors, for which we would not expect to see causal effects due to their temporal ordering (the outcome occurring before the exposure). These negative controls included the effect of AFB on age at menarche, AFS on age at menarche, AFB on AFS, age at menopause on AFS and age at last birth (ALB) on age at menarche. In these cases, any evidence of an effect would suggest pleiotropy. This may occur when a genetic instrument affects the exposure and outcome through a shared heritable factor, which could be a shared process or pathway [44].

#### Heterogeneity

We performed a test for heterogeneity using Cochran’s *Q* statistic using the TwoSampleMR package between instruments. A *Q* larger than the number of instruments minus one provides evidence for heterogeneity and invalid instruments, which can imply the presence of pleiotropy [45, 46].

#### Pleiotropy

We used additional MR methods: weighted mode [47], weighted median [48] and MR-Egger [49] to assess evidence of pleiotropy [50]. The intercept and 95% confidence interval of the MR-Egger regression line was used to determine directional pleiotropy using the TwoSampleMR package [49]. In addition, the *I*^*2*^ statistic was calculated to quantify the strength of the instruments used for MR-Egger to determine if the genetic variants violated the “NO Measurement Error” (NOME) assumption [51]. For IVs with an *I*^*2*^ statistic < 90%, we performed additional MR-Egger sensitivity analyses using the simulation extrapolation (SIMEX) to adjust for regression dilution bias.

We also applied the R function MR-PRESSO (Mendelian Randomisation Pleiotropy RESidual Sum and Outlier) to identify and correct for potential outliers (*p* < 0.05) [52]. Further details can be found in Additional file 1 [46–50, 52, 53].

#### Steiger filtering for bidirectional relationships

We performed the MR Steiger test and Steiger filtering bidirectionally for pairs of reproductive factors where the temporal ordering was not clear [54] (Additional file 2: Table S1). This was performed to assess whether the hypothesised causal directional of the relationship was correct for each genetic instrument [54]. Further details can be found in Additional file 1 [54].

### Assessing the impact of sample overlap

To investigate whether the degree of bias introduced by sample overlap impacted our findings, we conducted a series of sensitivity analyses.

Firstly, we performed MR on GWAS summary statistics using a “split-sample” approach, in which the UK Biobank sample was divided in two halves at random. The MR analysis was performed twice for each relationship, once using the exposure GWAS from one half and the outcome GWAS from the second half and vice versa, with the resulting MR effect estimates being meta-analysed using a fixed effects model.

Secondly, we performed two-sample MR using results from largely non-UK Biobank replication studies and consortia to estimate SNP effects on the exposure (sample 1) and UK Biobank estimates to estimate SNP effects on the outcome (sample 2), and vice versa where appropriate [31, 32, 55–58]. Further details on the number of studies and sample sizes used for the replication consortia are shown in Additional file 2: Table S3. Using replication studies may also avoid bias introduced by winner’s curse, which is the overestimation of SNP effects on the exposure in a discovery GWAS [59, 60].

Finally, we used a recently developed MR method, MRlap, that is robust to bias introduced by sample overlap, winner’s curse and weak instruments [61]. MRlap was performed using the UK Biobank GWAS summary statistics for reproductive factors where both the exposure and outcome were continuous, i.e. excluding associations involving ever parous status, as the correction for biases cannot account for a different degree of overlap for cases/controls in case of binary traits [61]. Further details can be found in Additional file 1 [42, 60–62].

Only those reproductive factor associations for which there was evidence of an effect from the primary analysis were taken forward for this sensitivity analysis, as the causal effect would likely be overestimated when performing MR with overlapping exposure and outcome samples [42].

### Evaluating the role of adiposity

Childhood adiposity may confound the relationships between reproductive factors that we identify since adiposity is genetically correlated with age at menarche [63], and age at menarche is genetically correlated with other reproductive factors [64, 65]. To investigate this, we performed multivariable MR (MVMR) using the “MVMR” R package, adjusting for childhood body size from UK Biobank [66].

## Results

### UK Biobank

**Fig. 1:**
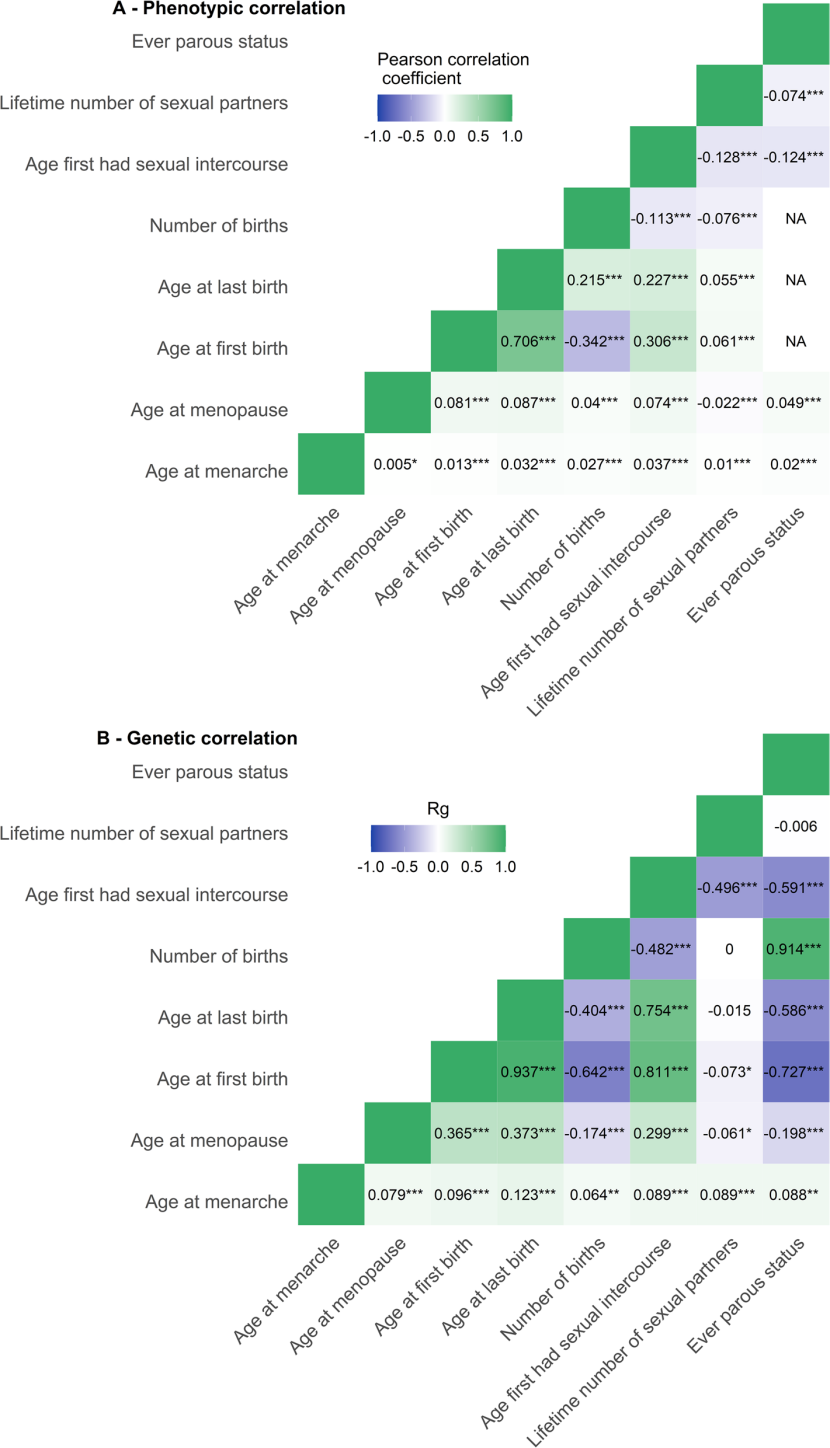
**A** Phenotypic correlation using the Pearson correlation coefficient. **B** Genetic correlation between reproductive factors using LD score regression. ***<0.001; **<0.01; *<0.05. The values in each correlation square are the Pearson correlation coefficient and Rg for **A** and **B** respectively

A total of 264,698 women from UK Biobank were included in this analysis. The mean age at assessment was 56.4 years (SD = 8.0); further sample characteristics are shown in Table 1. Many of the reproductive factors were weakly phenotypically correlated. The strongest correlations were between AFB and ALB (Pearson correlation coefficient = 0.71), and AFB and number of births (Pearson correlation coefficient = −0.34) (Fig. 1A).

**Table 1:** UK Biobank reproductive factor descriptives

**Reproductive factor**	***N***	**Mean (SD)**
Age at menarche (years)	243,898	13.0 (1.6)
Age first had sexual intercourse (years)	219,486	19.1 (3.6)
Age at first live birth (years)	203,606	25.9 (5.1)
Age at last live birth (years)	203,356	30.1 (5.2)
Age at menopause (years)	143,791	49.7 (5.1)
Number of live births	250,746	1.8 (1.2)
**Reproductive factor**	***N***	**Median (IQR)**
Lifetime number of sexual partners	208,274	3 (4)
**Reproductive factor**	**(% (*N*))**	
Never parous	18.69 (49,358)	

*SD* standard deviation, *IQR* interquartile range, *N* sample size

### UK Biobank GWAS

Table 2 displays the number of variants associated with the eight reproductive factors at genome-wide significance (*p* < 5 × 10^−8^) after LD clumping within the full UK Biobank sample. Between four (ever parous status) and 223 (age at menarche) SNPs were identified. All *F* statistics were above the standard threshold of 10, indicative of strong genetic instruments (Table 2).

**Table 2:** Sample size of the exposure (*N*), *F* statistic and the number of SNPs (nSNPs) used within the primary analysis

Exposure	*N*	*F* statistic	*R*^2^	nSNPs
Age at menarche	243,898	74.95 (75.28 for outcome AFS)	0.064	223 (222 for outcome AFS^a^)
Age at menopause	143,791	85.08	0.047	84
Age at first live birth	203,606	41.97	8.38 × 10^−3^	41
Age at last live birth	203,356	43.46	1.92 × 10^−3^	9
Number of live births	250,746	46.75	1.68 × 10^−3^	9
Age first had sexual intercourse	219,486	39.46	9.44 × 10^−3^	53
Lifetime number of sexual partners	208,274	39.20	6.36 × 10^−3^	34
Ever parous status	250,746	53.92	8.59 × 10^−4^	4

*AFS* age first had sexual intercourse. ^a^One palindromic SNP was excluded from the MR between age at menarche and AFS

### Genetic correlation

The LDSC revealed that the 8 reproductive factors were genetically correlated (*r*_g_ range: |0.06–0.94|), except for the lifetime number of sexual partners, which was not correlated with ALB, number of births or ever parous status (*r*_g_ < 0.01). Age at menarche was only weakly genetically correlated with other reproductive factors (*r*_g_ range: 0.06–0.12). In general, genetic correlations were larger in magnitude than the corresponding phenotypic correlations (Fig. 1B, Additional file 2: Table S4).

### Mendelian randomisation

#### Effect of age at menarche

**Fig. 2:**
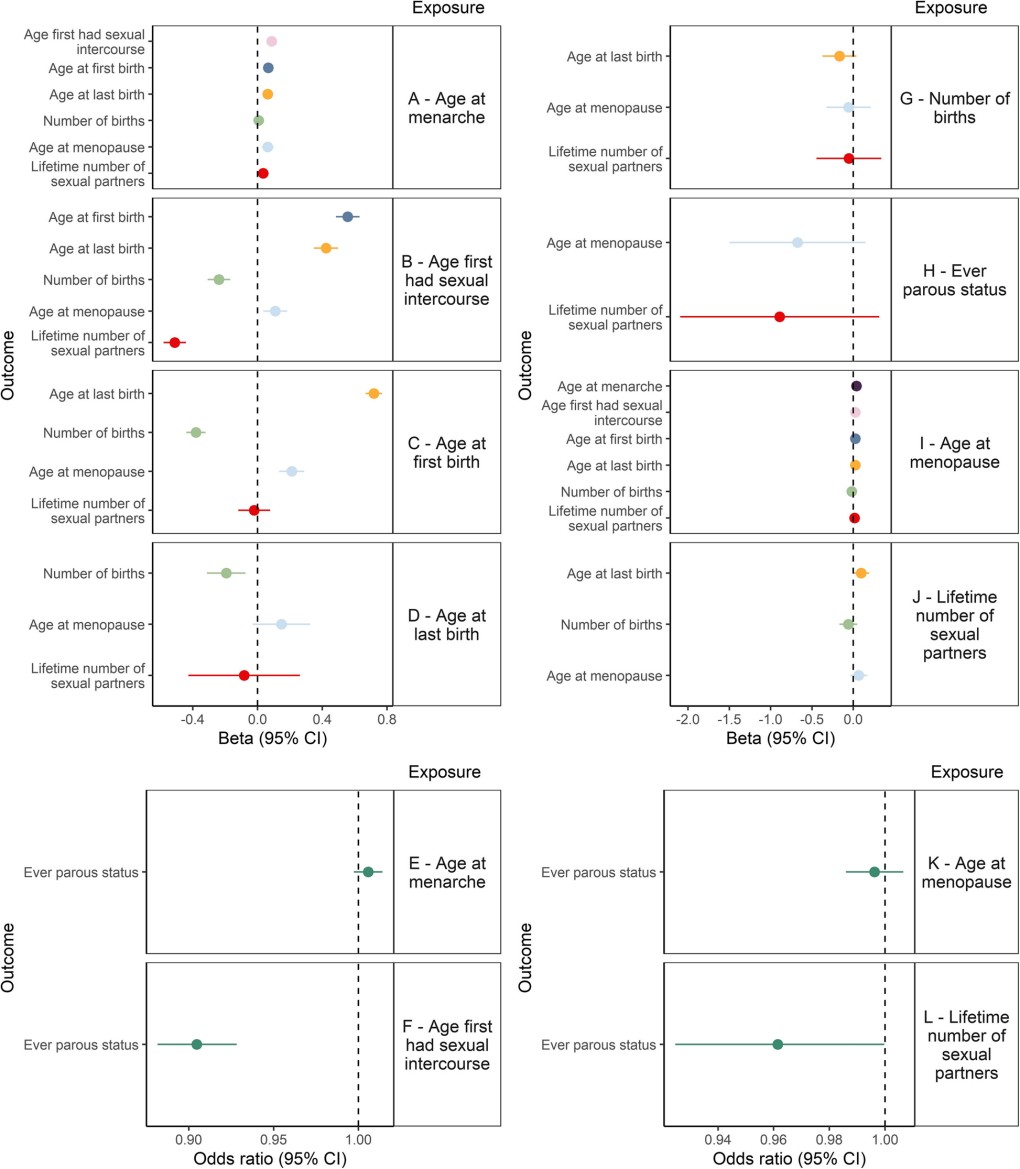
Mendelian randomisation of inter-relationships between UK Biobank reproductive factors. Panels to the right of plots **A**–**L** refer to the exposures investigated by MR, on outcomes shown on the *y* axis. GWAS summary statistics were standardised prior to performing MR

MR findings from the primary analysis suggest that the positive genetic correlation reflects a causal relationship between later age at menarche (1 SD increase) and later AFS (beta (*B*) = 0.09 SD, 95% confidence intervals (*CI*) = 0.06, 0.11), AFB (*B* = 0.07 SD, *CI* = 0.04, 0.10), ALB (*B* = 0.06 SD, *CI* = 0.04, 0.09) and age at menopause (*B* = 0.06 SD, *CI* = 0.03, 0.10) (Fig. 2A).

#### Effects of age first had sexual intercourse

In addition, later AFS (1 SD increase) appears to lead to later age at menopause (*B* = 0.11 SD, *CI* = 0.04, 0.18), later AFB (*B* = 0.56 SD, *CI* = 0.49, 0.63), later ALB (*B* = 0.42 SD, *CI* = 0.35, 0.50), lower number of births (*B* = −0.24 SD, *CI* = −0.31, −0.17), lower lifetime number of sexual partners (*B* = −0.51 SD, *CI* = −0.58, −0.44) and increased likelihood of not having any children (odds ratio (*OR*) = 0.90 SD, *CI* = 0.88, 0.93) (Fig. 2B, F).

#### Effect of age at first birth

Findings suggest later AFB (1 SD increase) may lead to a later age at menopause (*B* = 0.21 SD, *CI* = 0.13, 0.29), later ALB (*B* = 0.72 SD, *CI* = 0.67, 0.77) and lower number of births (*B* = −0.38 SD, *CI* = −0.44, −0.32) (Fig. 2C).

#### Effect of age at last birth

Findings suggest later ALB (1 SD increase) may lead to a lower number of births (*B* = −0.19 SD, *CI* = −0.31, −0.07) (Fig. 2D).

#### Effect of lifetime number of sexual partners

Finally, a higher lifetime number of sexual partners decreases the likelihood of having children (*OR* = 0.96 SD, *CI* = 0.92, 1.0) (Fig. 2L).

Number of births, ever having children and age at menopause do not appear to have strong effects on any of the other reproductive factors (Fig. 2G, H, I, K), although confidence intervals for the effects of number of births and ever having children are wide.

**Fig. 3:**
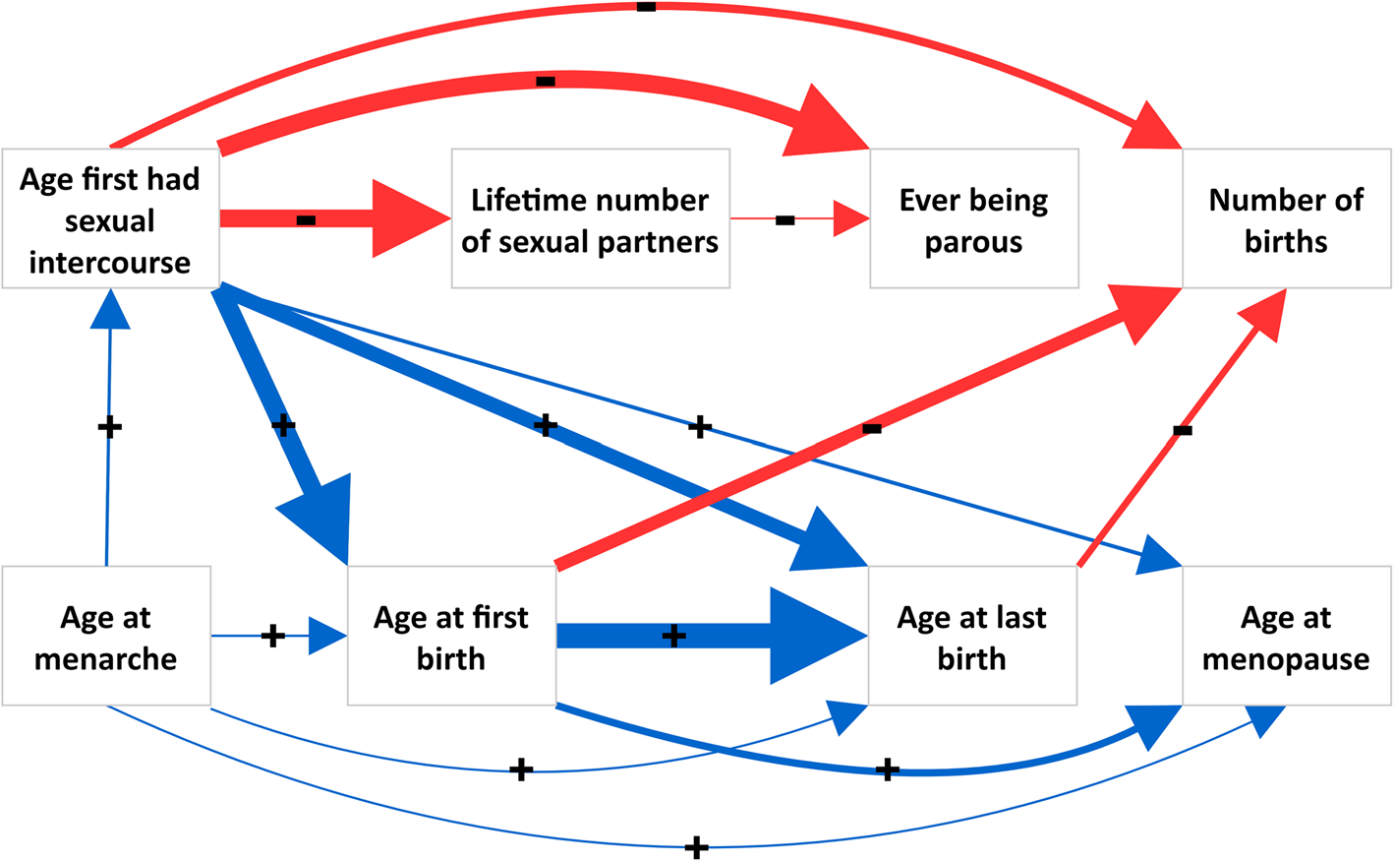
Relationships identified in the primary with evidence of a causal effect. The relationship between age at menarche and lifetime number of sexual partners is not highlighted here due to the attenuation of effect in the sensitivity analyses. + along with a green arrow indicates a positive relationship, and – along with a red arrow indicates a negative relationship. The weight of the arrows represents the relative magnitude of the effect

Full results of this analysis can be found in Additional file 2: Table S5, and a causal graph shows where we found evidence of an effect between reproductive factors (Fig. 3).

### Evaluating MR assumptions

#### Negative controls

We found little evidence for an effect of age at menopause on AFS (*B* = 0.03 SD, *CI* = −1.32 × 10^−3^, 0.05), of ALB on age at menarche (*B* = 0.11 SD, *CI* = −0.12, 0.34), of AFB on age at menarche (*B* = 0.04 SD, *CI* = −0.07, 0.16) or of AFS on age at menarche (*B* = 0.10 SD, *CI* = −7.03 × 10^−4^, 0.21) (Additional file 2: Table S6). However, there was strong evidence for an effect of AFB on AFS (*B* = 0.58 SD, *CI* = 0.52, 0.65), suggestive of shared pleiotropy. To assess whether the effect identified between AFB and AFS was due to shared pleiotropy via age at menarche, we performed MVMR using the “MVMR” R package, including age at menarche as an additional exposure. Adjusting for age at menarche did not attenuate the effect of AFB on AFS (Additional file 2: Table S6), suggesting shared pleiotropy is likely to occur via another pathway.

#### Heterogeneity

For the relationships identified in the primary analysis, evidence for heterogeneity in the individual SNP effects in the IVW was present across many of the investigated relationships, except for between AFB and ALB and between ALB and number of births (Additional file 2: Table S7). Evidence for heterogeneity could indicate the presence of SNP outliers which were investigated using MR-PRESSO (see the “Pleiotropy” section).

#### Pleiotropy

The effects of age at menarche on AFS, of AFS on AFB and ALB and of AFB on ALB and menopause and number of births were consistent across MR-Egger, weighted median and weighted mode that test for the presence of pleiotropy (Additional file 2: Table S8, Additional file 1: Fig. S1).

Effects were less consistent across the additional MR methods between age at menarche and AFB, ALB, menopause and lifetime number of sexual partners, as well as AFS and age at menopause, lifetime number of sexual partners, number of births and ever being parous.

Furthermore, the effect of AFB on age at menopause, ALB on number of births and lifetime number of sexual partners and ever being parous appeared inconsistent across the different MR methods.

In the primary analysis, the only instance where the MR-Egger intercept test revealed evidence for directional pleiotropy was in the relationship between age at menarche and lifetime number of sexual partners (Additional file 2: Table S9).

We assessed the heterogeneity in gene-exposure estimates, or *I*^2^_GX_. The *I*^2^_GX_ was > 97% in all analyses, suggesting MR-Egger is performing optimally (Additional file 2: Table S10).

We also applied MR-PRESSO to the UK Biobank full overlap GWAS to additionally test for evidence of pleiotropy and correct for outliers (Additional file 2: Table S11). MR-PRESSO revealed evidence for outliers in almost all tests, other than for the relationships between AFB and ALB. However, after outlier correction, there was little change in the strength of evidence in the IVW estimates (Additional file 2: Table S12).

We applied an MR Steiger method to assess whether we had captured the intended causal direction between reproductive factors where the causal direction was unclear. Findings show aggregated instruments have successfully captured the intended causal direction in all cases (Additional file 2: Table S13). Steiger filtering was also implemented to assess whether there were any individual SNPs that did not capture the intended causal direction, and results are displayed in Additional file 2: Table S14. Where instruments contained SNPs that did not capture the intended causal direction, MR analysis was then performed excluding those SNPs and the strength of evidence for the causal estimate using the IVW method did not change (Additional file 2: Table S15).

### Assessing the impact of sample overlap

#### UK biobank split-sample

In the split-sample GWAS within UK Biobank, between 1 and 101 SNPs were identified at genome-wide significance (*p* < 5 × 10^−8^) after LD clumping (*r*^2^ < 0.001 and a distance of 10,000 kb) (Additional file 2: Table S16). No SNPs were identified at genome-wide significance in relation to ALB and parous status in the GWAS performed on one of the UK Biobank split-samples; therefore, the split-sample MR was only conducted once when ALB or ever parous status was the exposure.

Where SNPs were identified in the split-sample analysis, *F* statistics were above the standard threshold of 10, indicative of strong genetic instruments (Additional file 2: Table S16). However, there was little overlap in the SNPs which surpassed genome-wide significance between sample 1 and sample 2, with 9 SNPs overlapping between samples for age at menarche and age at menopause but none for the other traits (Additional file 2: Table S17). A number of the SNPs identified in one of the samples of the split-sample GWAS were identified above the significance threshold but removed during LD clumping in the GWAS of the other sample, while other SNPs were just below the significance threshold or appeared not to be associated (Additional file 2: Table S17).

We performed MR for each relationship twice, i.e. MR of exposure in sample 1 on outcome in sample 2 and MR of exposure in sample 2 on outcome in sample 1. This was with the exception of the MR analyses when ALB and parous status were the exposure, which were assessed only once (Additional file 2: Table S18). We then meta-analysed findings between both samples, which showed limited evidence of heterogeneity between the causal estimates obtained from the split-sample MRs. Full results of the meta-analysis can be found in Additional file 2: Table S19.

#### Replication consortia

Inter-relations between the reproductive factors were also investigated using GWAS summary statistics from consortia studies which excluded UK Biobank. Sixty SNPs were identified at genome-wide significance (*p* < 5 × 10^−8^) for age at menarche (ReproGen) and 5 for AFB (SSGAC) (Additional file 2: Table S20). All *F* statistics were above the standard threshold of 10, indicative of strong genetic instruments (Additional file 2: Table S20). Full results of this analysis can be found in Additional file 2: Table S21. Estimates were consistent when using a larger replication GWAS from ReproGen for age at menopause, although the sample this GWAS was performed in had a large proportion of UK Biobank overlap (Additional file 2: Table S3).

#### MRlap UK biobank

MRlap was performed using the reproductive factor GWAS summary statistics for the full UK Biobank sample. This method identified slightly more variants at genome-wide significance (*p* < 5 × 10^−8^) after LD pruning (10,000kb, *r*^2^ = 0.001) compared to the main analysis. Between 11 (ALB and number of births) and 231 (age at menarche) were identified (Additional file 2: Table S22). MR estimates were largely similar to the primary analysis, although in some cases the effect size was slightly larger, including for the relationship between ALB and number of births. Full results of this analysis can be found in Additional file 2: Table S23.

#### Assessing evidence of causal effects across sensitivity analyses

**Fig. 4:**
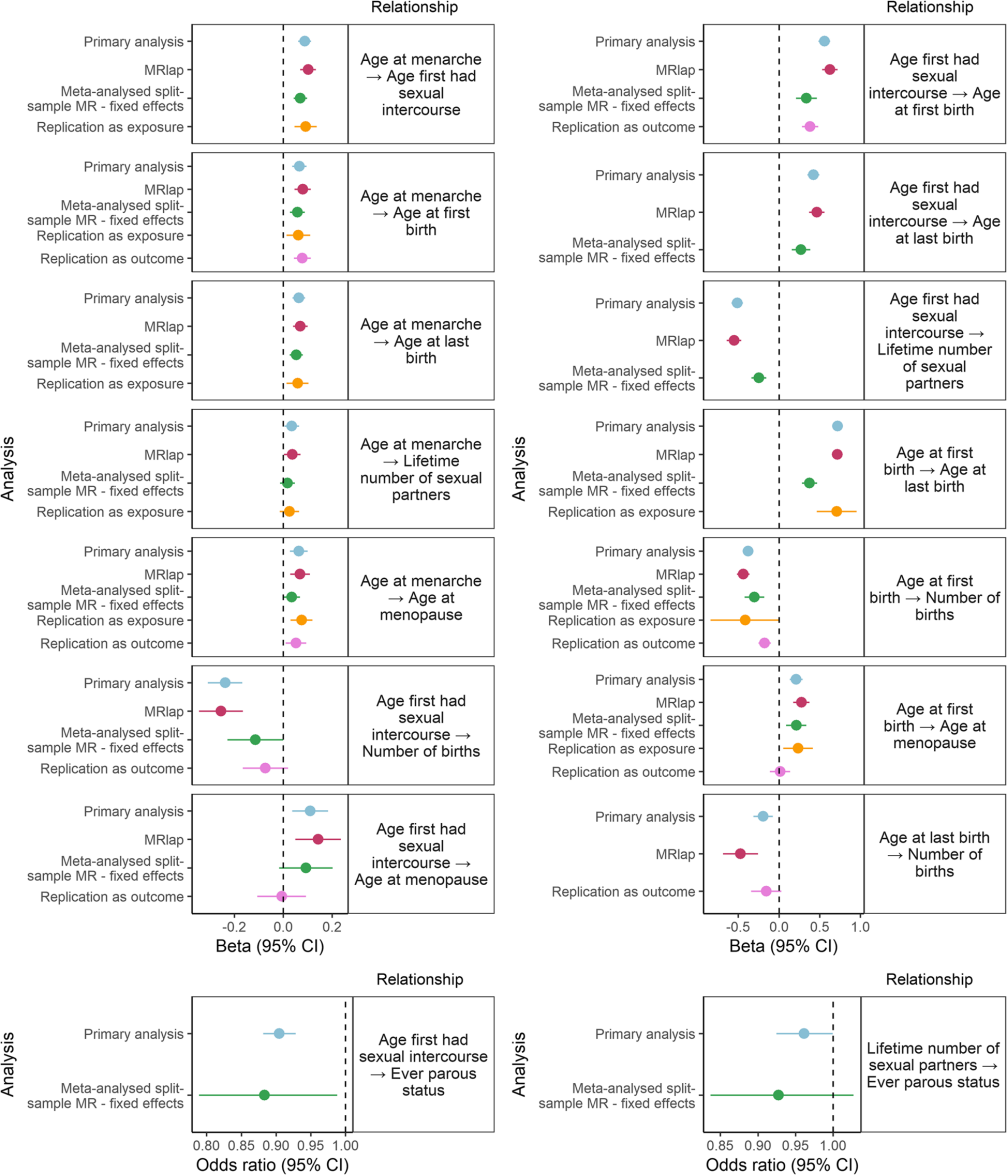
Mendelian randomisation estimates from the primary and across the sensitivity analyses. Panels to the right of the plots refer to the relationships investigated, and each analysis is shown on the y axis. All analyses were performed using the IVW MR method. GWAS summary statistics were standardised prior to performing MR

Figure 4 illustrates the effects which appear robust across multiple sensitivity analyses. In particular, a later age at menarche appears to have consistent effects on a later AFB, ALB and AFS. In addition, a later AFB leading to a later ALB, a later AFS leading to later AFB and a later AFS leading to a lower number of lifetime sexual partners were consistent across all sensitivity analyses. There was no consistent evidence for a causal relationship between age at menarche and lifetime number of sexual partners across sensitivity analysis and limited evidence between AFS and age at menopause.

### Evaluating the role of adiposity

We used MVMR analysis to adjust for childhood body size and assess for the presence of confounding. We found little evidence that adjustment of childhood body size impacts the relationships identified in the primary analysis. One exception was the relationship between age at menarche and lifetime number of sexual partners, which attenuates with adjustment for childhood body size, and which additional sensitivity analyses indicate may be affected by pleiotropy and bias induced by sample overlap (Additional file 2: Table S24).

## Discussion

This study provides evidence supporting causal effects of several female reproductive factors on other reproductive traits. We show evidence that earlier reproductive factors including age at menarche, AFS and AFB have effects on subsequent events and factors, while ever parous status, age at menopause, number of births, ALB and lifetime number of sexual partners appear to have limited effects on other reproductive factors.

We substantiate the genetic correlation between reproductive factors shown in previous studies, while showing additional correlations that have not been previously investigated [64, 65]. Our study supports evidence for a positive causal link between age at menarche and age at menopause [11–16, 67, 68] and opposes previous studies that have shown the inverse association [17, 18] or no association [19–24]. Furthermore, our findings support one study that found little evidence for an association between age at menarche and parity [26]. Additionally, we corroborated the findings of previous MR studies that identified a positive causal relationship between age at menarche and AFB, ALB and age at menopause, and between AFS and ALB [67–69].

Many estimates identified in the primary analysis appear consistent across sensitivity analyses that aim to account for biases. However, some results did not persist in sensitivity analyses checking for robustness to sample overlap and winner’s curse.

The split-sample meta-analysed MR shows a weaker magnitude of effect compared to our primary analysis, which may be due to sample size reduction in this sensitivity analysis or bias introduced by sample overlap in the primary analysis.

Overall, using replication GWAS studies as the exposure or outcome showed weaker strength of evidence and/or magnitude of effects, although evidence for a causal effect for many relationships assessed was maintained. This may be due to bias introduced by winner’s curse in the primary analysis or smaller sample sizes available for the replication studies. In particular, age at menopause from the ReproGen consortium has a sample size of 69,360, compared to 143,791 in our primary analysis, and where this is used as the outcome, we found little evidence of an effect of reproductive factors on age at menopause. A more recent GWAS of age at menopause conducted by the ReproGen consortium has a much larger sample size (*n* = 201,323) [70, 71], although more than half of the sample comprise UK Biobank women, meaning a large sample overlap in the MR analysis. Nonetheless, MR estimates using this more recent GWAS revealed similar results compared to the previous smaller GWAS [32]. While there are more recent, larger GWAS available for age at menarche [72] and AFB [73], UK Biobank has formed a large contribution to these GWAS. We decided to prioritise studies which had a smaller number of participants from UK Biobank for the replication GWAS, in order to reduce the likelihood of bias due to sample overlap.

The difference in how the phenotype for age at menopause between UK Biobank and ReproGen has been derived may contribute to differences in estimated effects. While both GWAS have excluded women who had a hysterectomy, ReproGen additionally excludes women who had a bilateral ovariectomy, those who had menopause induced by radiation or chemotherapy and those using hormone replacement therapy [70].

MRlap revealed almost identical results compared to our primary analysis suggesting sample overlap may not substantially bias estimates.

Pleiotropy may occur when genetic variants have an effect on multiple phenotypes, which can be an issue in MR as the genetic instruments used as a proxy for the exposure can affect the outcome independently of the exposure of interest [29, 60]. Therefore, resulting effect estimates may not correctly capture the exposure-outcome relationship of interest. This could be a problem as many of the reproductive factors are genetically correlated, and consequently, multiple sensitivity analyses were used to assess whether there was an exclusion restriction assumption violation. We implemented additional MR methods and numerous relationships did not appear to be affected by pleiotropy. Where outlier correction was possible, results were consistent with the primary analysis, with the exception of the effect of lifetime number of sexual partners on ever having children, where there was a complete attenuation of the effect after outlier correction.

However, it is worth considering that a recent study found that using MR-Egger on overlapping exposure and outcome samples may induce bias in the direction and magnitude of the confounding. This bias attenuates when the MR-Egger method is performing optimally, i.e. when it is employed with maximum variability in instrument strength. This is expressed as heterogeneity in gene-exposure estimates across SNPs, also referred to as *I*^*2*^_*GX*_, which can be calculated using the *I*^*2*^ statistic. It is estimated that the bias in MR-Egger when used in a one-sample setting is substantially reduced when *I*^*2*^_*GX*_ is higher than the recommended 90% [43]. Conversely, other two-sample methods appear to perform similarly in a one-sample MR compared to a two-sample approach in similarly large sample size [43]. Where there was evidence of non-null effects in the primary analysis, the *I*^*2*^_*GX*_ was >97% suggesting MR-Egger is performing optimally. Nonetheless, the MR-Egger test can be underpowered, especially when few instruments are available.

### Mechanisms underlying causal links

We show that an earlier age at menarche may lead to an earlier AFS and AFB, as well as an earlier AFS leading to an earlier AFB. It is likely that earlier maturation may lead to earlier sexual activity, logically increasing the chance of an earlier pregnancy. In UK Biobank, a proportion of women may have had first had sexual intercourse prior to the introduction of the NHS family planning act 1967 which made contraception readily available through the NHS. This may have strengthened the effect of AFS on AFB in this cohort and findings may not be generalisable to more contemporary studies. We also show that an earlier AFS may lead to a higher number of sexual partners, which may occur due to a longer amount of time to acquire partners if sexual activity commences earlier. Furthermore, we identify that having a higher lifetime number of sexual partners may lead to a lower chance of having children. This may be due to the increased prevalence of short-term relationships and regularly changing sexual partners [74], which, as a result, might lead to less chance of starting a family. However, it is worth noting that after excluding outlying variants, the effect between lifetime number of sexual partners and ever parous status attenuated. We present strong evidence for a positive relationship between AFB and ALB. One explanation for this link could be as parents tend to have children in a relatively short period of time, as shown in UK Biobank where the average AFB is 26 years, and ALB is 30 years for women.

The life history theory is another explanation as to why earlier age at menarche leads to earlier subsequent reproductive events and a likelihood of an increased number of children. This theory distinguishes the allocation of resources into growth and reproductive efforts and categorises “fast” or “slow” life history strategies [75, 76]. A “fast” life history strategy exerts more effort towards reproduction: earlier puberty and sexual activity leading to an early AFB, and an increased number of births [75, 76]. This is corroborated by our finding that women who experience an earlier AFS have children earlier and have more children. If a woman starts having children earlier, they have more opportunity to conceive again before menopause, which may explain the effect we identify between an earlier AFB and a higher number of children. A “fast” life history may lead to an earlier age at menopause as allocating resources towards reproductive efforts earlier in life and towards a higher number of children, which may result in a completing reproduction at a younger age.

There were a number of relationships where we did not find evidence for an effect in our primary analysis. Of note, we did not find a causal effect of age at menarche on the number of births and ever parous status. Considering the life history theory, we might have expected to find an inverse effect, suggesting an earlier age at menarche leads to a high number of births.

Furthermore, we did not find evidence of an effect of ever parous status on lifetime number of sexual partners and number of births on ALB. We investigated bidirectional effects between reproductive factors where there was not a clear temporal order and identified no bidirectional effects. Specifically, there were no effects between age at menopause and ALB, lifetime number of sexual partners, number of births and ever parous status, ALB and lifetime number of sexual partners and finally number of births and lifetime number of sexual partners.

Several relationships between reproductive factors separated by many years could be mediated by other intervening reproductive events. For example, we identify effects between age at menarche and AFS, AFS and AFB, and age at menarche and AFB; therefore, the effect we find between age at menarche and AFB may be mediated by AFS. Similarly, we found effects between AFS and AFB, AFB and ALB, and AFS and ALB, which could suggest that an earlier AFS leading to an earlier ALB may be mediated through an earlier AFB. In addition, there are likely to be mediating mechanisms for the relationships we have identified other than through reproductive factors such as body mass index [63]. Future investigations could use mediation analyses to further elucidate these relationships [77].

### Implication of findings

When investigating one reproductive factor in relation to a health outcome, our findings might aid in identifying reproductive factors that could confound this relationship. For example, becoming a parent at an earlier age has been identified as a risk factor for depressive symptoms in young adulthood [78, 79]. We have presented evidence that age at menarche has a causal effect on AFB, and previous studies have identified earlier age at menarche as a risk factor for poor mental health outcomes [80, 81]. The evidence presented in this study suggests it would be important to adjust for age at menarche in an investigation of the effects of AFB on mental health outcomes.

Our work also suggests that reproductive factors might lie on the causal pathway between an earlier reproductive factor and a later outcome. We present evidence for a causal effect between AFB and number of births, and both reproductive factors have been identified as a risk factor for cardiovascular disease [82]. An investigation of AFB on the risk of cardiovascular disease might want to consider mediation via the number of births.

Finally, a number of reproductive factors have been identified as risk factors for breast cancer, including age at menarche, age at menopause [2], number of births and AFB [3]. We have presented a number of causal inter-relationships between reproductive factors; therefore, researchers should carefully consider the total impact of reproductive factor variability on chronic diseases such as breast cancer rather than the impact of single reproductive indicators, and a multivariable approach could be particularly useful [83].

### Strengths and limitations

The strengths of the study include the range of reproductive factors investigated using the MR approach, the use of the large UK Biobank resource and data from other genetic consortia, and the extent of MR sensitivity analyses to evaluate MR assumptions and address sample overlap. However, this study has a number of limitations.

Firstly, negative control analysis revealed strong evidence of an effect of AFB on AFS, suggesting possible evidence of pleiotropy which has been previously identified for the AFS genetic instrument [84]. As this may reduce the reliability of our results, future work could further assess whether the associations identified for AFS reflect true causal effects.

For some exposures such as ALB, the number of births and ever being parous, the number of SNPs used as genetic instruments was limited, meaning we cannot reliably evaluate pleiotropy and heterogeneity in these instances. Increasing the number of SNPs in the genetic instruments for each of these reproductive factors through larger GWAS would be valuable.

Another limitation is the issue of selection bias in UK Biobank. While 9 million individuals were invited to participate in the study, the response rate was 5%. Additionally, the participants in the UK Biobank and replication studies we used were largely restricted to women of European ancestry. These samples are therefore not representative of the entire UK female population and estimates may not be generalisable to women in other ancestry groups. In addition, these findings may not be representative of younger generations of women considering the average age of UK Biobank participants, and the evidence of secular trends in some reproductive factors. For example, there is evidence that there is a long-term downward trend in age at menarche [85] and increase in AFB [86]. Future work is required to replicate our findings in contemporary independent studies and translate the results in women in other ancestry groups.

While the majority of the reproductive factors are likely to be accurately captured through a questionnaire (such as AFB, number of births and ALB), other factors such as age at menarche may not be as reliably recalled [87]. Self-report of lifetime number of sexual partners is also known to be overestimated by some, which could explain the positively skewed distribution we identified [88]. To account for this, we performed a rank-based inverse normal transformation of this variable.

It is also worth noting that some reproductive events may not have been fully captured in the analysis, as certain reproductive milestones may not have been reached by some women. For example, younger women who were reported to have not had children may subsequently have children. In addition, the ALB and number of births may not reflect final reproductive milestones if some women go on to have more children. However, considering the mean age of UK Biobank women is 56.4 years (SD = 8.0), there are likely to be few women who go on to have more children.

The split-sample GWAS revealed little overlap between genome-wide significant SNPs identified in each sample. While some of these SNPs were identified slightly below the significance threshold between samples, others appeared not to be associated. This suggests that some SNPs may have been identified through spurious associations and may suggest evidence of winner’s curse.

## Conclusion

In conclusion, we present evidence of inter-relationships between reproductive factors. In particular, we find strong evidence of an effect of age at menarche, AFS and AFB on subsequent reproductive events and factors. Future work should consider the inter-relationships between reproductive factors when assessing reproductive risk on disease outcomes.

## Supplementary Information

**Additional file 1: Figure S1.** Forest plots showing effect estimates of additional MR methods for relationships identified in the primary MR analysis. Panels A-P refer to the relationships assessed using MR, and MR methods used is shown on the y axis.

**Additional file 2: Table S1** Relationships where bi-directional MR was performed due to unclear temporal ordering. **Table S2** Relationships investigated. **Table S3** Replication consortia and studies. **Table S4** Genetic correlation results. **Table S5** Primary analysis (IVW). **Table S6** Negative control results. **Table S7** Heterogeneity for primary analysis. **Table S8** Additional MR methods in relation to primary analysis. **Table S9** Egger intercept test for the primary analysis. **Table S10** I-squared statistics. **Table S11** MR PRESSO Global test for primary analysis. **Table S12** MR PRESSO Outlier correction for primary analysis. **Table S13** Steiger results for the primary analysis. **Table S14** Steiger: SNPs found to be in the incorrect intended for the primary analysis direction. **Table S15** Steiger filtered MR results for the primary analysis. **Table S16** Split sample SNPs, R2, F stats and number of overlapping SNPs. **Table S17** Split sample GWAS overlapping SNPs between samples. **Table S18** IVW UKBB split sample results. **Table S19** UKBB meta-analysed split sample results. **Table S20** Replication SNPs, R^2^ and F stats. **Table S21** IVW UKBB and replication results. **Table S22** MRlap number of SNPs. **Table S23** MRlap observed and corrected results. **Table S24** MVMR findings adjusted for childhood body size (UK Biobank).

## Data Availability

The availability of all data analysed in this study has been referenced throughout the manuscript and supplementary materials. https://www.reprogen.org/ https://www.thessgac.org/

## Abbreviations

AFB: Age at first birth
AFS: Age first had sexual intercourse
ALB: Age at last birth
*B*: Beta
CI: Confidence interval
GWAS: Genome-wide association studies
IEU: Integrative Epidemiology Unit
IVW: Inverse variance weighted
LD: Linkage disequilibrium
LDSC: Linkage disequilibrium score regression
MR: Mendelian randomisation
MRC: Medical Research Council
MR-PRESSO: Mendelian Randomisation Pleiotropy RESidual Sum and Outlier
OR: Odds ratio
SD: Standard deviation
SNP: Single nucleotide polymorphism
