# Supplementary figures and images for: The relationships between women’s reproductive factors: a Mendelian randomisation analysis
Source: BMC Med. 2022 Mar 24;20:103. doi: 10.1186/s12916-022-02293-5 (PMC8944090; doi:10.1186/s12916-022-02293-5)

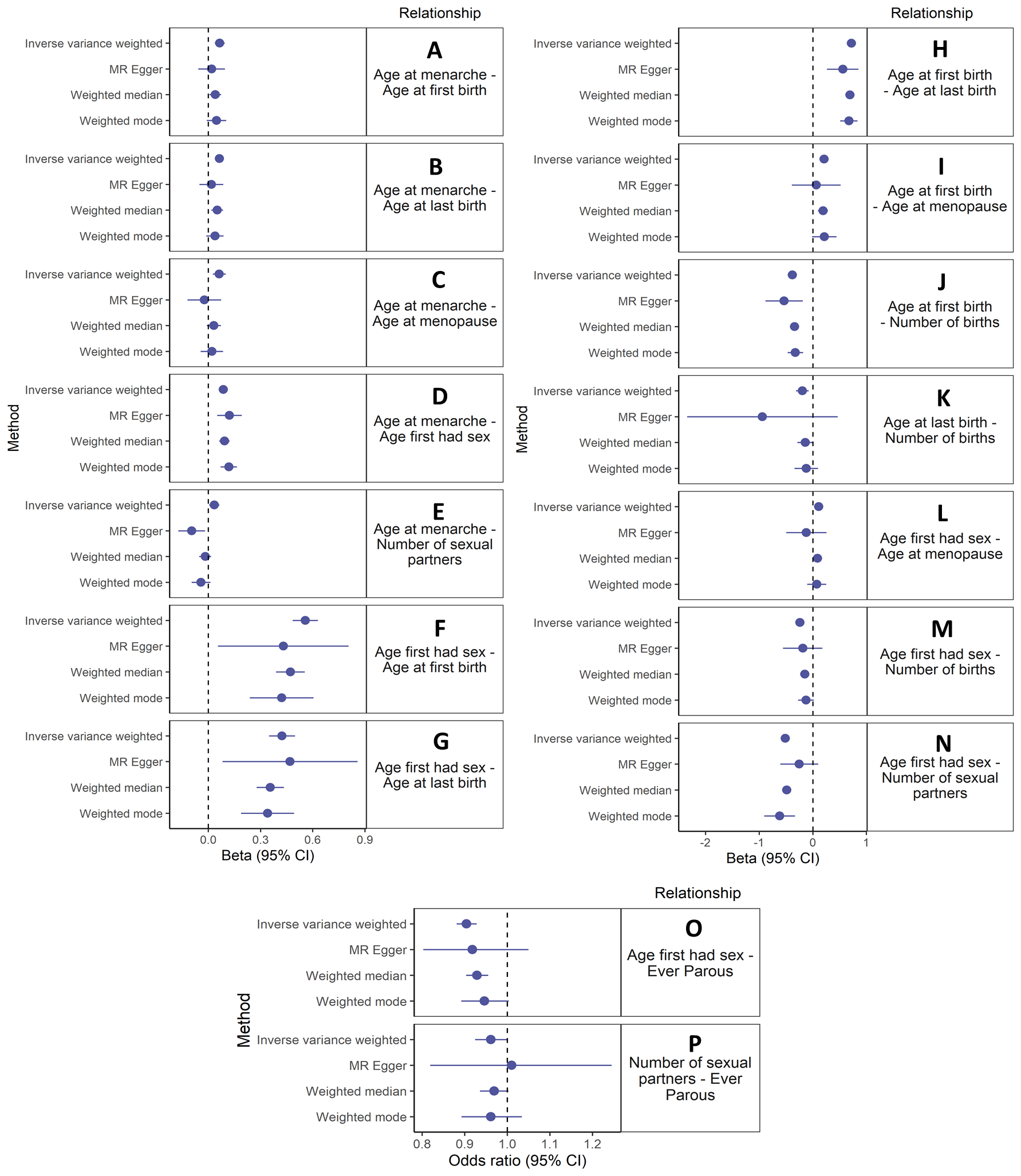

Supplement: Supplementary file 1 — Additional file 1: Figure S1. Forest plots showing effect estimates of additional MR methods for relationships identified in the primary MR analysis. Panels A-P refer to the relationships assessed using MR, and MR methods used is shown on the y axis. [file 12916_2022_2293_MOESM1_ESM.zip › Figure S1R2.tiff]
